# Association between trunk-to-peripheral fat ratio and renal function in elderly Japanese men: baseline data from the Fujiwara-kyo Osteoporosis Risk in Men (FORMEN) study

**DOI:** 10.1265/ehpm.22-00279

**Published:** 2023-05-13

**Authors:** Katsuyasu Kouda, Yuki Fujita, Chikako Nakama, Kumiko Ohara, Takahiro Tachiki, Junko Tamaki, Akiko Yura, Jong-Seong Moon, Etsuko Kajita, Nami Imai, Kazuhiro Uenishi, Masayuki Iki

**Affiliations:** 1 Department of Hygiene and Public Health, Kansai Medical University, 2-5-1 Shin-machi, Hirakata, Osaka 573-1010, Japan; 2 Center for Medical Education, Kindai University Faculty of Medicine, 377-2 Oono-higashi, Osaka-Sayama, Osaka 589-8511, Japan; 3 Chukyo Gakuin University Faculty of Nursing, 2216 Tokicho, Mizunami, Gifu 509-6192, Japan; 4 Department of Hygiene and Public Health, Faculty of Medicine, Osaka Medical and Pharmaceutical University, 2-7 Daigakumachi, Takatsuki, Osaka 569-8686, Japan; 5 Department of Public Health, Kindai University Faculty of Medicine, 377-2 Oono-higashi, Osaka-Sayama, Osaka 589-8511, Japan; 6 Department of Nursing, Kio University, 4-2-2 Umami-naka, Koryo-cho, Nara 635-0832, Japan; 7 Department of Pharmaceutical and Medical Business Sciences, Nihon Pharmaceutical University, 10281 Komuro, Ina-machi, Kitaadachi-gun, Saitama 362-0806, Japan; 8 Laboratory of Physiological Nutrition, Kagawa Nutrition University, 3-9-21 Chiyoda, Sakado, Saitama 350-0288, Japan

**Keywords:** Adiposity, Densitometry, Epidemiology, Kidney

## Abstract

**Background:**

Central obesity as measured by waist-to-hip circumference ratio (WHR) has been reported to be associated with renal hemodynamics and function. However, the adipose component of WHR, which is a composite measure of fat mass and fat-free mass, is small, particularly in nonobese subjects. Trunk-to-peripheral fat ratio as measured using dual-energy absorptiometry (DXA) is a more precise method for evaluating central fat distribution than WHR. The present study investigated the cross-sectional association between DXA-measured trunk-to-peripheral fat ratio and estimated glomerular filtration rate (eGFR) in community-dwelling elderly Japanese men.

**Methods:**

Participants were 575 men aged ≥65 years at the time of the baseline survey of the second Fujiwara-kyo Osteoporosis Risk in Men (FORMEN) cohort study. Trunk-to-appendicular fat ratio (TAR) was calculated as trunk fat divided by appendicular fat (sum of arm and leg fat), and trunk-to-leg fat ratio (TLR) as trunk fat divided by leg fat.

**Results:**

eGFR values significantly decreased from the lowest to the highest quintile of TAR/TLR. After adjusting for potential confounding factors including whole-body fat, the highest quintile of both TAR and TLR showed statistically significant odds ratios for the risk of eGFR <60 ml/min/1.73 m^2^, relative to the lowest quintile. In addition, a significant decreasing trend was observed for eGFR values from the lowest to the highest quintile of TAR/TLR after adjusting for confounding factors including whole-body fat.

**Conclusion:**

Elderly men with a large trunk-to-peripheral fat ratio tended to have a lower eGFR. This association occurred independently of that between whole-body fat and eGFR.

## Background

Obesity is associated with the pathogenesis of type 2 diabetes, hypertension, dyslipidemia, cardiovascular diseases, cancer, and kidney diseases [1]. Central obesity as measured by waist-to-hip circumference ratio (WHR) has been reported to be associated with renal hemodynamics and function [2, 3]. A large-scale observation study in the Netherlands reported that not only overweight and obese subjects but also lean subjects with a high WHR are at risk for renal function impairment [4]. Another study of a population of nonhypertensive, nondiabetic, healthy individuals suggested that a higher WHR, independent of body mass index (BMI), is associated with an unfavorable pattern of renal hemodynamic measures [5]. These studies suggest that central obesity is an independent risk factor for renal damage. However, the currently available epidemiological information is based on WHR, which is a composite measure of fat mass and fat-free mass, and the adipose component of WHR is small, particularly in nonobese subjects [6]. For example, WHR in lean subjects is influenced by gluteofemoral muscle mass.

Dual-energy absorptiometry (DXA) is used widely for clinical assessment of a patient’s risk of osteoporosis and increasingly to measure body composition in terms of fat and fat-free mass [7]. DXA has become recognized for its accurate and precise measurement of total body composition [8]. Trunk-to-peripheral fat ratio measured using DXA, which is a specific surrogate for visceral fat proportion, more precisely reflects central fat distribution than WHR [9]. Associations between DXA-measured trunk fat proportion and cardiometabolic risk factors have been reported for Europeans [10–12], North Americans [13, 14], and Asians [15, 16]. A higher trunk-to-peripheral fat ratio, independent of whole-body fat volume, is associated with an unfavorable pattern of cardiometabolic measures [16]. Therefore, DXA-measured central fat distribution may serve as a clinically useful parameter for risk evaluation of cardiometabolic syndrome [12]. In light of accumulating evidence of the association between cardiometabolic syndrome and chronic kidney disease [17], DXA-measured central fat distribution may be clinically useful in preventing chronic kidney disease. However, no data are available on the association between DXA-measured central fat distribution and renal function. Investigation of DXA-measured central fat distribution and renal function is needed. The present study aimed to investigate the cross-sectional association between DXA-measured trunk-to-peripheral fat ratio and estimated glomerular filtration rate (eGFR) in community-dwelling elderly Japanese men.

## Methods

### Study participants

The present cross-sectional study used baseline data from the second Fujiwara-kyo Osteoporosis Risk in Men (FORMEN) cohort study [16]. The FORMEN study is a community-based single-center prospective cohort study that aims to determine risk factors for osteoporotic fractures and increase the number of healthy life years in elderly Japanese men. The FORMEN study consists of the first (baseline survey from June 2007 to October 2008) and second (baseline survey from August 2019 to January 2020) cohorts. Details of the first cohort study have been described elsewhere [18]. Briefly, participants were men aged ≥65 years at the time of the baseline survey who lived at home in four cities located in Nara Prefecture, were able to walk without the assistance of another individual, and were able to provide self-reported information and written informed consent. DXA-measured central fat distribution was measured at the baseline survey in the second, but not in the first cohort study. The next follow-up survey in the second cohort study is scheduled for 2025. In the second cohort study, 599 elderly men participated in the baseline survey. After excluding 24 men who either had missing values for DXA-measured central fat distribution or blood test, or who had a medical history of kidney disease including renal cell carcinoma, 575 participants were included in the present analysis.

### Measurement of whole-body fat and central fat distribution

Whole-body fat and central fat distribution were assessed using the same DXA scanner (QDR-4500A; Hologic Inc., Bedford, MA, USA) mounted on a mobile examination car as described previously [15]. Quality control checks of DXA equipment were performed on a regular basis using several Hologic phantoms. Intra-machine variation for whole-body fat calculated from 11 measurements with 2 volunteers was 3.0% (coefficient of variation). A multicenter study using QDR 4500 devices reported an inter-instrumental variation of 5.6% (coefficient of variation) for whole-body fat [19]. Measurements for all participants were performed by the same experienced medical radiology technician. Before the DXA scan, participants were asked to change into an examination gown, remove all metal objects (e.g., accessories, watches, zippers, belts, underwire bras) and their shoes, and were asked to lie down on their back on the scanning table without a pillow and keep still during the examination, and whole-body posterior-anterior (PA) scan images were obtained. Head, arm, and leg regions in whole-body PA scan images were separated from the trunk region using manufacturer-recommended methods [7]: (a) head separation using the horizontal neck (shoulder) line just below the chin; (b) arm separation using the vertical shoulder line bisecting the shoulder joints; and (c) leg separation using the lower pelvic divider lines (two angled lines) bisecting both femoral necks. Fat mass was measured separately for the arm, trunk, and leg regions. Appendicular fat mass was calculated using arm fat mass and leg fat mass. Trunk-to-appendicular fat ratio (TAR) was calculated as trunk fat divided by appendicular fat, and trunk-to-leg fat ratio (TLR) as trunk fat divided by leg fat. Fat mass index was calculated as whole-body fat divided by height squared (kg/m^2^) [20].

### Measurements of body size and blood pressure

Body weight and height were measured in light clothing with no shoes. Body mass index (BMI) was calculated as weight divided by height squared (kg/m^2^). Blood pressure was measured using an automated device (BP-203i, OMRON COLIN, Tokyo, Japan) with an appropriate cuff size. Participants were relaxed and seated with legs uncrossed in a quiet room with comfortable temperature. Measurements were performed with the right mid-arm at the heart level after resting for 5 minutes. The mean value of two readings was used for analysis.

### Biochemical analysis

Blood samples were obtained by vein puncture after an overnight fast, allowed to clot, and centrifuged. Levels of serum creatinine (mg/dl) were determined by the enzymatic method (Determiner L CRE, Hitachi Chemical Diagnostics Systems Co., Ltd., Tokyo Japan). eGFR (ml/min/1.73 m^2^) was calculated using the Modification of Diet in Renal Disease Study modified for Japanese individuals by the Japanese Society of Nephrology, as follows: eGFR = 194 × serum creatinine^−1.094^ × age^−0.287^ [21]. Cutoff levels of eGFR followed a guideline from the National Institute for Health and Care Excellence [22].

### Medical history and lifestyle factors

To identify potential confounding factors affecting the relationships between TAR/TLR (predictors) and eGFR (outcome), we obtained information on medical history and lifestyle factors including smoking status, physical activity, and dietary intake [23]. Participants completed a questionnaire survey that covered medical history including malignant diseases, hypertension, diabetes mellitus, kidney disease, and medications to treat these diseases. Participants were also asked to bring current prescriptions of medications to the baseline survey, and trained healthcare nurses recorded the names and doses of the medications. Moreover, the nurses interviewed participants based on their answers to a questionnaire that covered lifestyle factors including physical activity and smoking (current or non-smoker). Physical activity levels were determined using the Japanese version of the International Physical Activity Questionnaire (IPAQ), which has been validated for Japanese adults aged 65 years and older in the Fujiwara-kyo study [24]. Metabolic equivalent of task (MET)-minutes/week was calculated in accordance with official IPAQ guidelines [25]. Dietary intake of nutrients was estimated using the food frequency questionnaire (FFQ) for dietary intake of nutrients in Japan (FFQ for the Prevention and Management of Osteoporosis; FFQPOP [26]). Participants were asked to select a food intake frequency grade during the past one month using the FFQ. Selected frequency or amount of food was also confirmed by dietitians during their interviews with participants. Nutrient and energy intake amounts per day were calculated using data listed in the Standard Table of Food Composition in Japan [27].

### Definitions of hypertension and diabetes

Participants with hypertension were defined as those with systolic blood pressure ≥140 mmHg and/or diastolic blood pressure ≥90 mmHg following repeated examination [28] and/or the use of antihypertensive drugs. Participants with diabetes were defined as those with hemoglobin A1c (National Glycohemoglobin Standardization Program) ≥6.5% [29] and/or the use of antidiabetic drugs.

### Statistics

Data were analyzed using SPSS Statistics Desktop for Japan, Version 26 (IBM Japan, Ltd., Tokyo, Japan). A P-value less than 0.05, or 95% confidence interval not containing the value 1, was considered statistically significant. Linear and nonlinear regression models were used to analyze the association between TAR/TLR and eGFR. Pearson’s correlation coefficients or Spearman’s rank correlation coefficients were used to assess associations between quintiles of TAR/TLR (predictor) and other variables, and between categories of eGFR (outcome) and other variables. To obtain an adjusted odds ratio (aOR), multivariate logistic regression analysis was used after adjusting for potential confounders that may affect associations between TAR/TLR (predictors) and eGFR (outcome). The logistic regression analysis incorporated potential confounders including variables that could be theorized, such as hypertension and diabetes mellitus. In addition to the theorized variables, those associated with both eGFR (outcome) and TAR/TLR (predictors) at a P-value <0.2 were used in the logistic regression analysis. Duplicate variables such as weight, BMI, whole-body fat, fat mass index, and body fat percentage were excluded from the same multivariate analysis. To assess linear trends of eGFR values from the lowest to the highest quintile of TAR/TLR, multivariate regression analysis was performed after adjusting for potential confounders. The dependent variable was eGFR value, and independent variables were potential confounding factors and TAR/TLR quintile.

## Results

**Fig. 1:**
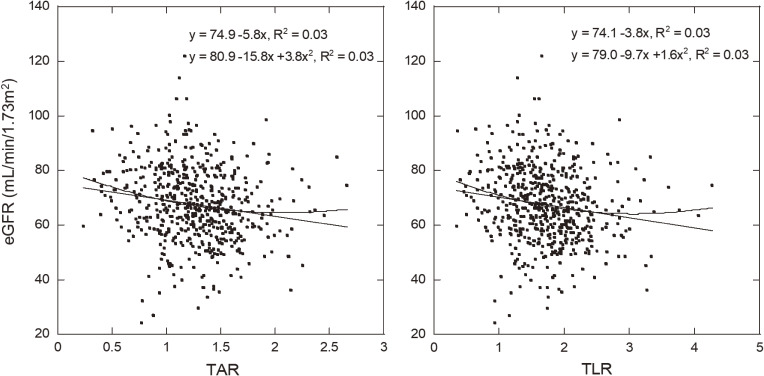
Associations between trunk-to-peripheral fat ratio and estimated glomerular filtration rate. TAR, trunk-to-appendicular fat ratio; TLR, trunk-to-leg fat ratio; eGFR, estimated glomerular filtration rate. Linear and polynomial regression models were used to analyze the association between TAR/TLR and eGFR.

Table 1 shows the characteristics of analyzed subjects, including age, anthropometry, physical activity, smoking habit, alcohol consumption, nutritional intake, medical history, DXA-measured parameters, and eGFR. Figure 1 shows the associations between TAR/TLR and eGFR obtained from the linear and nonlinear regression models. R^2^ of the nonlinear (polynomial) regression model was similar to that of the linear regression model. Subgroup characteristics stratified by quintile values of TAR/TLR and cutoff levels of eGFR are shown in Tables 2 and 3, respectively. eGFR values significantly decreased from the lowest to the highest quintile of TAR/TLR, and other variables such as age, anthropometry, medical history, whole-body fat, and fat-free soft tissue mass showed significant associations with TAR/TLR (Table 2). TAR/TLR values significantly increased from the ≥90 eGFR group to the >45 eGFR group, and other variables such as age, anthropometry, hypertension, and whole-body fat showed significant associations with eGFR (Table 3). eGFR showed no significant association with MET-minutes/week/100, smoking habit, alcohol intake, energy intake, or protein intake (Table 3). After stratification by MET-minutes/week/100 or energy intake, we observed significant associations between TAR/TLR and eGFR (TAR in MET below median, r = −0.20; TAR in MET above median, r = −0.12; TLR in MET below median, r = −0.19; TLR in MET above median, r = −0.13; TAR in energy below median, r = −0.19; TAR in energy above median, r = −0.13; TLR in energy below median, r = −0.18; TLR in energy above median, r = −0.14). Table 4 shows aORs for the risk of eGFR <60 ml/min/1.73 m^2^ in each quintile of TAR/TLR relative to the lowest quintile in total participants (575 men). In every model (Models 1, 2, and 3), aORs in the highest quintile were statistically significant, and the ratio of aOR for TLR to aOR for TAR in Model 3 was 1.18 in the highest quintile. In addition, eGFR values showed a significant trend to decrease from the lowest to the highest quintile of TAR/TLR after adjusting for confounding factors including whole-body fat in every model. Table 5 shows aORs for the risk of eGFR <60 ml/min/1.73 m^2^ in each quintile of TAR/TLR relative to the lowest quintile in participants with BMI <25 kg/m^2^ (433 men without over weight). In every model (Models 1, 2, and 3), aORs in the highest quintile were statistically significant. In addition, eGFR values showed a significant trend to decrease from the lowest to the highest quintile of TAR/TLR after adjusting for confounding factors including whole-body fat in every model.

**Table 1:** Participant characteristics

	**Overall, N = 575**	
Age (years), mean (SD)	73.3	(5.4)
Height (cm), mean (SD)	165	(6)
Weight (kg), mean (SD)	63.2	(8.4)
BMI (kg/m^2^), mean (SD)	23.2	(2.7)
MET-minutes/week, median (25, 75%ile)	2310	(1059, 4533)
Current smoker, N (%)	52	(9)
Alcohol intake (kcal/day), mean (SD)	93	(139)
Energy intake (kcal/day), mean (SD)	1710	(310)
Protein intake (g/day), mean (SD)	72	(16)
Hypertension, N (%)	394	(69)
Diabetes, N (%)	121	(21)
BMI ≥25 kg/m^2^, N (%)	142	(25)
BMI <18.5 kg/m^2^, N (%)	23	(4)
Whole-body fat (kg), mean (SD)	12.9	(4.7)
Body fat percentage (%), mean (SD)	21.0	(5.8)
Fat mass index (kg/m^2^), mean (SD)	4.8	(1.7)
Fat-free soft tissue mass (kg), mean (SD)	45.2	(4.8)
eGFR (mL/min/1.73 m^2^), mean (SD)	67.6	(13.5)

N, number; SD, standard deviation; BMI, body mass index;

MET, metabolic equivalent of task; eGFR, estimated glomerular filtration rate.

**Table 2:** Subgroup characteristics stratified by quintile values of TAR or TLR

	**TAR**										**P-value^a^**

	**Q1 (0.23–0.96)**		**Q2 (0.96–1.18)**		**Q3 (1.18–1.34)**		**Q4 (1.34–1.54)**		**Q5 (1.54–2.66)**		
Age (years), mean (SD)	75.0	(6.0)	72.7	(5.2)	72.5	(4.7)	73.8	(5.5)	72.5	(4.9)	0.01
Height (cm), mean (SD)	164	(6)	166	(6)	165	(6)	165	(6)	165	(5)	0.57
Weight (kg), mean (SD)	56.7	(7.4)	62.7	(8.5)	64.1	(7.8)	67.2	(8.5)	65.3	(5.9)	<0.01
BMI (kg/m^2^), mean (SD)	21.1	(2.2)	22.9	(2.8)	23.6	(2.6)	24.7	(2.4)	24.0	(2.0)	<0.01
MET-minutes/week, median (25, 75%ile)	2266	(990, 5592)	2400	(1032, 5172)	2640	(1188, 4746)	2358	(1074, 4326)	2064	(990, 4053)	0.35
Current smoker, N (%)	9	(8)	14	(12)	12	(10)	4	(3)	13	(11)	0.84
Alcohol intake (kcal/day), mean (SD)	96	(169)	69	(100)	68	(91)	109	(134)	121	(173)	0.03
Energy intake (kcal/day), mean (SD)	1719	(325)	1723	(323)	1741	(298)	1719	(307)	1647	(292)	0.11
Protein intake (g/day), mean (SD)	70	(16)	73	(15)	73	(15)	72	(17)	70	(15)	0.89
Hypertension, N (%)	73	(63)	77	(67)	75	(65)	85	(74)	84	(73)	0.06
Diabetes, N (%)	16	(14)	23	(20)	22	(19)	28	(24)	32	(28)	<0.01
BMI >25 kg/m^2^, N (%)	5	(4)	19	(17)	35	(30)	45	(39)	38	(33)	<0.01
BMI <18.5 kg/m^2^, N (%)	14	(12)	4	(3)	3	(3)	1	(1)	1	(1)	<0.01
Whole-body fat (kg), mean (SD)	8.7	(3.3)	12.8	(5.0)	14.0	(4.5)	15.2	(4.1)	13.9	(3.3)	<0.01
Body fat percentage (%), mean (SD)	15.8	(5.1)	21.0	(6.2)	22.5	(5.5)	23.6	(4.8)	22.3	(4.0)	<0.01
Fat mass index (kg/m^2^), mean (SD)	3.3	(1.2)	4.7	(1.9)	5.1	(1.6)	5.6	(1.4)	5.1	(1.2)	<0.01
Fat-free soft tissue mass (kg), mean (SD)	43.6	(5.4)	44.9	(4.8)	45.0	(4.3)	46.3	(5.3)	46.0	(3.8)	<0.01
eGFR (mL/min/1.73 m^2^), mean (SD)	69.9	(13.7)	70.8	(15.2)	67.5	(12.8)	64.8	(13.1)	64.9	(11.7)	<0.01

	**TLR**										**P-value^a^**

	**Q1 (0.35–1.25)**		**Q2 (1.26–1.56)**		**Q3 (1.56–1.82)**		**Q4 (1.82–2.13)**		**Q5 (2.13–4.27)**		

Age (years), mean (SD)	75.0	(6.1)	72.9	(5.1)	72.8	(5.1)	73.2	(5.3)	72.6	(4.8)	<0.01
Height (cm), mean (SD)	164	(7)	166	(5)	165	(7)	165	(6)	165	(5)	0.63
Weight (kg), mean (SD)	56.5	(7.3)	63.7	(9.0)	64.1	(7.8)	66.2	(8.1)	65.4	(6.1)	<0.01
BMI (kg/m^2^), mean (SD)	21.0	(2.2)	23.1	(3.0)	23.6	(2.6)	24.4	(2.4)	24.0	(1.9)	<0.01
MET-minutes/week, median (25, 75%ile)	2700	(1120, 5244)	2593	(1230, 4590)	1866	(924, 3657)	2720	(1188, 5238)	2133	(990, 4158)	0.26
Current smoker, N (%)	8	(7)	14	(12)	12	(10)	9	(8)	9	(8)	0.76
Alcohol intake (kcal/day), mean (SD)	90	(160)	72	(113)	77	(96)	103	(138)	122	(170)	0.02
Energy intake (kcal/day), mean (SD)	1741	(344)	1701	(314)	1730	(274)	1717	(312)	1660	(300)	0.11
Protein intake (g/day), mean (SD)	71	(17)	71	(14)	72	(14)	73	(17)	70	(16)	0.84
Hypertension, N (%)	73	(63)	77	(67)	75	(65)	85	(74)	84	(73)	0.06
Diabetes, N (%)	19	(17)	20	(17)	23	(20)	26	(23)	33	(29)	0.01
BMI >25 kg/m^2^, N (%)	5	(4)	26	(23)	31	(27)	45	(39)	35	(30)	<0.01
BMI <18.5 kg/m^2^, N (%)	14	(12)	4	(3)	2	(2)	2	(2)	1	(1)	<0.01
Whole-body fat (kg), mean (SD)	8.7	(3.4)	13.5	(5.5)	13.7	(4.0)	14.9	(4.3)	13.8	(3.2)	<0.01
Body fat percentage (%), mean (SD)	15.9	(5.1)	21.7	(6.5)	22.1	(5.0)	23.5	(5.2)	22.0	(3.9)	<0.01
Fat mass index (kg/m^2^), mean (SD)	3.3	(1.2)	4.9	(2.0)	5.0	(1.4)	5.5	(1.5)	5.1	(1.2)	<0.01
Fat-free soft tissue mass (kg), mean (SD)	43.4	(5.2)	45.2	(4.7)	45.3	(4.8)	45.8	(4.9)	46.2	(4.1)	<0.01
eGFR (mL/min/1.73 m^2^), mean (SD)	70.2	(13.7)	69.8	(14.6)	68.0	(14.4)	65.2	(12.0)	64.7	(12.1)	<0.01

TAR, trunk-to-appendicular fat ratio; TLR, trunk-to-leg fat ratio; Q, quintile; SD, standard deviation; BMI, body mass index;

MET, metabolic equivalent of task; N, number; eGFR, estimated glomerular filtration rate.

^a^Trend analysis using Pearson’s correlation coefficient or Spearman’s rank correlation coefficient.

**Table 3:** Subgroup characteristics stratified by cutoff levels of eGFR

	**eGFR (mL/min/1.73 m^2^)**								**P-value^a^**

	**≥90**		**<90, ≥60**		**<60, ≥45**		**<45**		
			
**N**	**27**		**380**		**143**		**25**		
Age (years), mean (SD)	71.9	(5.0)	72.8	(5.2)	74.1	(5.3)	78.3	(5.9)	<0.01
Height (cm), mean (SD)	163	(5)	165	(6)	165	(5)	163	(7)	0.67
Weight (kg), mean (SD)	58.0	(7.4)	63.2	(8.3)	64.0	(8.0)	64.3	(11.4)	0.01
BMI (kg/m^2^), mean (SD)	21.8	(3.3)	23.2	(2.7)	23.5	(2.4)	24.0	(3.3)	<0.01
MET-minutes/week, median (25, 75%ile)	1617	(594, 5652)	2510	(1074, 4770)	2064	(1230, 4068)	1800	(693, 3100)	0.15
Current smoker, N (%)	4	(15)	37	(10)	11	(8)	0	(0)	0.09
Alcohol intake (kcal/day), mean (SD)	105	(129)	95	(144)	93	(137)	48	(69)	0.22
Energy intake (kcal/day), mean (SD)	1711	(357)	1706	(310)	1715	(303)	1736	(309)	0.66
Protein intake (g/day), mean (SD)	73	(19)	71	(15)	71	(16)	74	(19)	0.79
Hypertension, N (%)	19	(70)	248	(65)	105	(73)	22	(88)	0.03
Diabetes, N (%)	7	(26)	77	(20)	30	(21)	7	(28)	0.82
BMI ≥25 kg/m^2^, N (%)	3	(11)	94	(25)	36	(25)	9	(36)	0.18
BMI <18.5 kg/m^2^, N (%)	4	(15)	15	(4)	3	(2)	1	(4)	0.04
Whole-body fat (kg), mean (SD)	10.7	(5.1)	12.8	(4.5)	13.5	(4.5)	14.6	(6.0)	<0.01
Body fat percentage (%), mean (SD)	18.8	(6.7)	20.8	(5.7)	21.8	(5.7)	23.2	(7.0)	<0.01
Fat mass index (kg/m^2^), mean (SD)	4.1	(2.1)	4.7	(1.6)	4.9	(1.6)	5.4	(2.1)	<0.01
Fat-free soft tissue mass (kg), mean (SD)	42.7	(3.5)	45.3	(4.8)	45.3	(4.7)	44.3	(6.1)	0.56
TAR, mean (SD)	1.09	(0.33)	1.24	(0.38)	1.36	(0.35)	1.25	(0.33)	<0.01
TLR, mean (SD)	1.47	(0.52)	1.68	(0.58)	1.85	(0.53)	1.68	(0.53)	<0.01

eGFR, estimated glomerular filtration rate; N, number; SD, standard deviation; BMI, body mass index;

MET, metabolic equivalent of task; TAR, trunk-to-appendicular fat ratio; TLR, trunk-to-leg fat ratio.

^a^Linear trend analysis using Pearson’s correlation coefficient or Spearman’s rank correlation coefficient.

**Table 4:** aOR of eGFR <60 ml/min/1.73 m^2^ in total participants, N = 575

	**aOR^a^**	**95% CI**	**P for trend^b^**		**aOR^a^**	**95% CI**	**P for trend^b^**
Model 1							
TAR, Q2/Q1	1.17	(0.61, 2.27)	<0.01	TLR, Q2/Q1	1.51	(0.77, 2.93)	<0.01
TAR, Q3/Q1	1.32	(0.69, 2.55)		TLR, Q3/Q1	1.42	(0.73, 2.76)	
TAR, Q4/Q1	2.03	(1.07, 3.86)		TLR, Q4/Q1	2.28	(1.19, 4.39)	
TAR, Q5/Q1	2.39	(1.26, 4.51)		TLR, Q5/Q1	2.80	(1.47, 5.33)	
Age, 1 SD increase	1.57	(1.20, 2.06)		Age, 1 SD increase	1.61	(1.23, 2.12)	
Hypertension, present/absent	1.43	(0.94, 2.17)		Hypertension, present/absent	1.42	(0.94, 2.17)	
Diabetes, present/absent	1.02	(0.65, 1.60)		Diabetes, present/absent	1.02	(0.65, 1.60)	
Fat mass index, 1 SD increase	1.10	(0.82, 1.49)		Fat mass index, 1 SD increase	1.09	(0.81, 1.47)	
Model 2							
TAR, Q2/Q1	1.18	(0.61, 2.28)	<0.01	TLR, Q2/Q1	1.51	(0.78, 2.95)	<0.01
TAR, Q3/Q1	1.33	(0.69, 2.56)		TLR, Q3/Q1	1.43	(0.73, 2.77)	
TAR, Q4/Q1	2.03	(1.06, 3.88)		TLR, Q4/Q1	2.29	(1.18, 4.43)	
TAR, Q5/Q1	2.39	(1.26, 4.53)		TLR, Q5/Q1	2.80	(1.47, 5.36)	
Age, 1 SD increase	1.57	(1.20, 2.06)		Age, 1 SD increase	1.61	(1.23, 2.12)	
Hypertension, present/absent	1.42	(0.94, 2.17)		Hypertension, present/absent	1.42	(0.93, 2.17)	
Diabetes, present/absent	1.02	(0.65, 1.60)		Diabetes, present/absent	1.02	(0.65, 1.61)	
Body fat percentage, 1 SD increase	1.10	(0.81, 1.48)		Body fat percentage, 1 SD increase	1.08	(0.80, 1.46)	
Model 3							
TAR, Q2/Q1	1.24	(0.64, 2.38)	<0.01	TLR, Q2/Q1	1.59	(0.82, 3.09)	<0.01
TAR, Q3/Q1	1.40	(0.73, 2.68)		TLR, Q3/Q1	1.48	(0.77, 2.87)	
TAR, Q4/Q1	2.12	(1.12, 4.03)		TLR, Q4/Q1	2.38	(1.24, 4.58)	
TAR, Q5/Q1	2.51	(1.33, 4.71)		TLR, Q5/Q1	2.91	(1.54, 5.52)	
Age, 1 SD increase	1.62	(1.22, 2.15)		Age, 1 SD increase	1.66	(1.25, 2.20)	
Hypertension, present/absent	0.98	(0.71, 1.35)		Hypertension, present/absent	0.96	(0.70, 1.33)	
Diabetes, present/absent	1.45	(0.95, 2.21)		Diabetes, present/absent	1.44	(0.95, 2.21)	
Height, 1 SD increase	1.01	(0.64, 1.59)		Height, 1 SD increase	1.02	(0.65, 1.60)	
Whole body fat, 1 SD increase	0.99	(0.73, 1.34)		Whole body fat, 1 SD increase	0.98	(0.72, 1.33)	
Fat-free soft tissue mass, 1 SD increase	1.12	(0.81, 1.54)		Fat-free soft tissue mass, 1 SD increase	1.13	(0.82, 1.55)	

aOR, adjusted odds ratio; eGFR, estimated glomerular filtration rate; N, number;

CI, confidence interval; TAR, trunk-to-appendicular fat ratio; Q, quintile; TLR, trunk-to-leg fat ratio; SD, standard deviation.

^a^aOR using binomial logistic regression analysis after adjusting for potential confounders shown below.

^b^P for linear trend of eGFR values from Q1 to Q5 using regression analysis after adjusting for potential confounders shown below.

Model 1, adjusted for age, hypertension, diabetes mellitus, and fat mass index.

Model 2, adjusted for age, hypertension, diabetes mellitus, and body fat percentage.

Model 3, adjusted for age, hypertension, diabetes mellitus, height, whole-body fat, and fat-free soft tissue mass.

**Table 5:** aOR of eGFR <60 ml/min/1.73 m^2^ in participants with BMI <25 kg/m^2^, N = 433

	**aOR^a^**	**95% CI**	**P for trend^b^**		**aOR^a^**	**95% CI**	**P for trend^b^**
Model 1							
TAR, Q2/Q1	1.84	(0.85, 4.02)	<0.01	TLR, Q2/Q1	1.68	(0.78, 3.62)	<0.01
TAR, Q3/Q1	1.16	(0.49, 2.73)		TLR, Q3/Q1	1.30	(0.57, 2.98)	
TAR, Q4/Q1	3.72	(1.64, 8.42)		TLR, Q4/Q1	2.57	(1.16, 5.73)	
TAR, Q5/Q1	3.57	(1.58, 8.06)		TLR, Q5/Q1	3.21	(1.45, 7.12)	
Age, 1 SD increase	1.42	(1.04, 1.95)		Age, 1 SD increase	1.43	(1.04, 1.95)	
Hypertension, present/absent	1.74	(1.06, 2.86)		Hypertension, present/absent	1.71	(1.04, 2.80)	
Diabetes, present/absent	0.97	(0.56, 1.66)		Diabetes, present/absent	0.93	(0.55, 1.59)	
Fat mass index, 1 SD increase	0.73	(0.42, 1.28)		Fat mass index, 1 SD increase	0.81	(0.47, 1.40)	
Model 2							
TAR, Q2/Q1	1.70	(0.79, 3.67)	<0.01	TLR, Q2/Q1	1.57	(0.73, 3.37)	<0.01
TAR, Q3/Q1	1.04	(0.45, 2.41)		TLR, Q3/Q1	1.19	(0.53, 2.67)	
TAR, Q4/Q1	3.26	(1.48, 7.17)		TLR, Q4/Q1	2.32	(1.06, 5.05)	
TAR, Q5/Q1	3.15	(1.43, 6.95)		TLR, Q5/Q1	2.90	(1.33, 6.33)	
Age, 1 SD increase	1.41	(1.03, 1.93)		Age, 1 SD increase	1.42	(1.04, 1.93)	
Hypertension, present/absent	1.71	(1.04, 2.81)		Hypertension, present/absent	1.68	(1.03, 2.76)	
Diabetes, present/absent	0.98	(0.57, 1.69)		Diabetes, present/absent	0.95	(0.56, 1.61)	
Body fat percentage, 1 SD increase	0.90	(0.58, 1.39)		Body fat percentage, 1 SD increase	0.96	(0.62, 1.47)	
Model 3							
TAR, Q2/Q1	1.92	(0.89, 4.16)	<0.01	TLR, Q2/Q1	1.76	(0.82, 3.77)	<0.01
TAR, Q3/Q1	1.17	(0.51, 2.71)		TLR, Q3/Q1	1.36	(0.61, 3.04)	
TAR, Q4/Q1	3.89	(1.76, 8.61)		TLR, Q4/Q1	2.69	(1.23, 5.86)	
TAR, Q5/Q1	3.67	(1.66, 8.10)		TLR, Q5/Q1	3.32	(1.53, 7.24)	
Age, 1 SD increase	1.42	(1.02, 1.98)		Age, 1 SD increase	1.42	(1.03, 1.97)	
Hypertension, present/absent	0.85	(0.58, 1.26)		Hypertension, present/absent	0.85	(0.58, 1.24)	
Diabetes, present/absent	1.77	(1.07, 2.93)		Diabetes, present/absent	1.72	(1.05, 2.84)	
Height, 1 SD increase	0.98	(0.57, 1.68)		Height, 1 SD increase	0.94	(0.55, 1.60)	
Whole body fat, 1 SD increase	0.65	(0.38, 1.10)		Whole body fat, 1 SD increase	0.72	(0.43, 1.22)	
Fat-free soft tissue mass, 1 SD increase	1.20	(0.78, 1.87)		Fat-free soft tissue mass, 1 SD increase	1.17	(0.76, 1.80)	

aOR, adjusted odds ratio; eGFR, estimated glomerular filtration rate; BMI, body mass index; N, number;

CI, confidence interval; TAR, trunk-to-appendicular fat ratio; Q, quintile; TLR, trunk-to-leg fat ratio; SD, standard deviation.

^a^aOR using binomial logistic regression analysis after adjusting for potential confounders shown below.

^b^P for linear trend of eGFR values from Q1 to Q5 using regression analysis after adjusting for potential confounders shown below.

Model 1, adjusted for age, hypertension, diabetes mellitus, and fat mass index.

Model 2, adjusted for age, hypertension, diabetes mellitus, and body fat percentage.

Model 3, adjusted for age, hypertension, diabetes mellitus, height, whole-body fat, and fat-free soft tissue mass.

## Discussion

The present study is the first to report on the association between eGFR and central fat distribution as precisely measured by DXA. In the present community-based single-center cross-sectional study among Japanese elderly men, a significant inverse association between eGFR and trunk-to-peripheral fat ratio was observed. Elderly men with a large distribution of central fat tended to have a lower eGFR. Moreover, this association remained significant after adjusting for potential confounding factors including whole-body fat. These findings suggest that the association between central fat distribution and eGFR is independent of the association between whole-body fat and eGFR. Central fat distribution as measured by DXA is an important determinant of renal function in this population. Not only overweight and obese subjects, but also subjects with central fat distribution, are at risk for renal impairment. DXA-measured trunk-to-peripheral fat ratio may serve as a clinical parameter that may aid in the prevention of chronic renal failure.

A previous cross-sectional study in the Netherlands reported that not only obese subjects but also lean subjects with a high WHR were at risk for renal function impairment [4]. Another study of healthy persons found that a higher WHR was associated with an unfavorable renal hemodynamic profile, lower glomerular filtration rate, lower effective renal plasma flow, and higher filtration fraction, even after adjusting for BMI [5]. The Study of Health in Pomerania reported a higher risk of microalbuminuria in subjects with a high WHR [30]. These results suggest that central fat distribution is an independent risk factor for renal damage. However, currently available epidemiological information is based on anthropometry such as WHR, which is influenced by gluteofemoral muscle mass. Therefore, to understand the association between central fat distribution and renal function more clearly, the present study investigated the association between DXA-measured trunk-to-peripheral fat ratio and eGFR. In so doing, we observed a significant inverse association between trunk-to-peripheral fat ratio and eGFR.

The pathophysiological background underlying the association between trunk-to-peripheral fat ratio and eGFR, independent of whole-body fat, was not explored in the present study. However, individuals who are not obese on the basis of height and weight can, like people with overt obesity, be hyperinsulinemic, insulin-resistant, and predisposed to type 2 diabetes, hypertriglyceridemia, and premature coronary heart disease [31]. Such metabolically obese, normal-weight (MONW) individuals are very common in the general population [31]. Most MONW individuals with relatively low BMI likely have significant excess visceral adipose tissue [32]. Increased visceral adipose tissue leads to renal glomerular hyperfiltration and hyperperfusion, which may lead to glomerular hypertrophy, proteinuria, and kidney disease [1]. Trunk fat measured by DXA consists of visceral and subcutaneous fat, while arm or leg fat does not include visceral fat. Trunk-to-peripheral fat ratio is a parameter of the relative amount of visceral adipose tissue and serves as a weight-independent index, and may provide additional information in the evaluation of health risks. Indeed, associations of DXA-measured TAR/TLR with adiponectin [33] and cardiometabolic status [15, 16] have been reported; both are independent of whole-body fat.

Although WHR is the most common parameter related to central fat distribution for all age groups, it is not a direct measure of the proportion of body fat, but rather a composite measure of fat mass and fat-free tissue mass; the adipose component of WHR is small, particularly in nonobese subjects [6]. Increased WHR may be a sign of higher waist circumference (reflecting increased visceral fat), reduced hip circumference (reflecting low gluteal muscle mass and/or low peripheral fat mass), or a combination of these [34]. In order to improve the evaluation and detection of central fat distribution, investigators and clinicians may need to employ some of the more accurate methodologies available today. It is important to measure fat or fat-free mass directly in several body parts to determine body fat distribution. Recently, techniques such as computed tomography, DXA, bioelectrical impedance analysis (BIA), and ultrasound imaging have been developed for the assessment of body composition and fat distribution. BIA is a simple, rapid, and commonly available technique used in both clinical practice and research studies. The recently developed equipment for BIA has been reported to allow for estimation of fat mass, fat-free mass, and muscle mass for the right arm, right leg, left arm, left leg, and trunk [35]. However, the precision and accuracy of BIA have yet to be determined [36, 37]. Meanwhile, the validity of BIA for assessing body composition was investigated by using DXA as a reference in previous studies [36, 37]. Therefore, we used DXA to assess trunk-to-peripheral fat ratio in the present study, although DXA is expensive.

The present study has some limitations worth noting. First, participants were not randomly selected; the study population was made up of people who met the entry criteria and were accessible to the investigators. Because some participants were more likely than others to volunteer, they are not fully representative of the Japanese elderly population. Older adults who volunteer or engage in more hours of volunteering have been reported to show higher levels of well-being [38]. In addition, present study subjects were only elderly males, and no females were examined. Thus, caution must be exercised when generalizing the results of this study. Second, the cross-sectional design did not allow us to establish causal relationships between central fat distribution and renal function. There is a possibility of the presence of a common cause for central fat distribution and renal function. In addition, we cannot investigate the temporal relation between outcomes and risk factors, and cannot deny the possibility that high TAR/TLR is a result of low eGFR. Third, GFR was estimated using serum creatinine in the present study, while other calculation methods, such as GFR estimated using cystatin C, was not performed. It is known that serum creatinine is influenced by muscle mass. Therefore, we used multivariate analysis after adjustment for fat free soft tissue mass.

## Conclusion

DXA-measured trunk-to-peripheral fat ratio, a parameter of central fat distribution, was associated with eGFR in elderly Japanese men. Elderly men with a large distribution of central fat tended to have a lower eGFR. Moreover, the observed association was independent of the association between whole-body fat and eGFR. Fat ratio parameters may be useful for predicting renal function, particularly in underweight to normal-weight populations. Trunk-to-peripheral fat ratios may allow for characterization of MONW individuals who are common in the general population. DXA-measured central fat distribution may serve as a clinical parameter that may aid in the prevention of chronic kidney disease in elderly men.

## Abbreviations

aOR: adjusted odds ratio
BIA: bioelectrical impedance analysis
BMI: body mass index
CI: confidence interval
DXA: dual-energy X-ray absorptiometry
eGFR: estimated glomerular filtration rate
FFQ: food frequency questionnaire
FFQPOP: FFQ for the Prevention and Management of Osteoporosis
FORMEN: Fujiwara-kyo Osteoporosis Risk in Men
IPAQ: International Physical Activity Questionnaire
MET: metabolic equivalent of task
MONW: metabolically obese, normal-weight
N: number
PA: posterior-anterior
Q: quintile
SD: standard deviation
TAR: trunk-to-appendicular fat ratio
TLR: trunk-to-leg fat ratio
WHR: waist-to-hip circumference ratio

## Declarations

### Availability of data and materials

The data that support the findings of the current paper are available from the corresponding author, [KK], upon reasonable request.
